# Racialised experience of detention under the Mental Health Act: a photovoice investigation

**DOI:** 10.1136/bmjment-2025-301655

**Published:** 2025-08-05

**Authors:** Kamaldeep Bhui, Roisin Mooney, Doreen Joseph, Rose McCabe, Karen Newbigging, Paul McCrone, Raghu Raghavan, Frank Keating, Nusrat Husain, Co Pact Research Group, Tara Morey

**Affiliations:** 1 Department of Psychiatry, University of Oxford, Oxford, UK; 2 City University, London, UK; 3 University of Birmingham—Edgbaston Campus, Birmingham, UK; 4 University of Greenwich, London, England, UK; 5 De Montfort University, Leicester, England, UK; 6 Law & Criminology, Royal Holloway University of London, Egham, UK; 7 The University of Manchester, Manchester, Manchester, UK

**Keywords:** Adult psychiatry, Schizophrenia & psychotic disorders

## Abstract

**Background:**

The rates of compulsory admission and treatment (CAT) are rising in mental health systems in the UK. Persistent disparities have been reported among migrants, and black and ethnic minorities in Europe and North America for decades. Lived experience data can provide novel insights to reduce coercive care.

**Methods:**

We purposively sampled people within 2 years of receiving CAT, to maximise diversity by age, sex, ethnicity and different ‘sections’ of the Mental Health Act (England and Wales) from eight health systems in England. Using participatory photovoice workshops, we assembled images, captions and reflective narratives, which were transcribed and subjected to thematic and intersectional analyses. The interpretation privileged lived experiences of participants and peer researchers alongside the research team. Preventive insights informed a logic model to reduce CAT.

**Results:**

Forty-eight ethnically diverse people contributed over 500 images and 30 hours of recorded narratives. A significant proportion of participants reported multimorbidity, adverse childhood experiences and carer roles. Their experiences indicated insufficient co-ordination to prevent CAT despite early help seeking; they were not taken seriously or believed when seeking help. Dismissive responses and even hostility from professionals and unnecessary police involvement were distressing, stigmatising and risked criminalisation. Participants wanted more (a) advocacy given in crisis, (b) trauma-informed therapeutic and creative support from inpatient into community settings, (c) family and carer involvement and (d) more information about how to negotiate care options, appeals, restriction and seclusion. Practitioners were felt to lack the essential skills to care for racialised and traumatised people subjected to CAT.

**Conclusions:**

We propose a lived experience logic model for the practice, policy and legislative solutions to reduce epistemic injustice, CAT and criminalising care.

**WHAT IS ALREADY KNOWN ON THIS TOPIC**

- Escalating rates of compulsory admission and treatment (CAT) and racial and ethnic disparities in CAT are well established in the UK, European and North American countries. People from racial and ethnic groups are under-represented in research, and so their voices do not enter policy or practice. This might be why no actions have led to reducing inequalities. Legislative, policy and practice reforms require better lived experience data, including those from racial and ethnic groups.

**WHAT THIS STUDY ADDS**

- We found epistemic injustice and racialisation in care processes, a lack of preventive actions despite help-seeking, dismissal of preventive efforts by patients, excessive use of police and coercive care and insufficient attention to culturally, racially and religiously competent assessment and care. People exposed to CAT have significant levels of adverse childhood experiences and multimorbidity requiring greater support in research and care processes. New skills standards are needed for people receiving CAT, including trauma-informed inpatient and aftercare, new standards for levels and methods of providing information and care during crises and skills on de-escalation. Advocacy, advanced agreements and discharge planning could be a vehicle for trauma-informed preventive interventions. We show new ways of undertaking inclusive research and methods for peer research and lived experience research in all aspects of study design, delivery and commissioning of research.

**IMPLICATIONS OF THE STUDY FOR RESEARCH, POLICY AND PRACTICE**

- The findings expose epistemic and structural mechanisms for persistent inequalities in care services. We set out preventive opportunities and make recommendations for practice, policy and legislation, specifically in England and Wales. We developed a logic model of how coercive care and CAT could be prevented. The findings are instructive for actions care sectors and more widely, to promote inclusive research.

## Background

 Black and ethnic minorities and migrants experience more barriers to accessing effective care and receive more coercive mental healthcare; for example, higher rates of compulsory admission and treatment (CAT), more contact with criminal justice agencies and poorer long-term mental health outcomes compared with White British people in the UK.^1^ These data reflect the picture in North America and Europe.^2^,^4^ Most explanations for these disparities focus on individual characteristics rather than care systems^5^; for example, traumatic events and psychosocial adversities such as early separation from parents, discrimination, unemployment, poverty and *gender violence*. Substance use, variations in judgements about risk and dangerousness and poor therapeutic alliances may also contribute. Escalating levels of CAT may also follow delays in accessing services because stigma might delay help-seeking, and there may also be fears of harms and coercion.^6^ The higher rates of CAT may reflect less support from carers or primary care.^7^ In addition, austerity measures led to reduced service capacity to prevent CAT.^8^ An Independent Review of the Mental Health Act (MHA, England & Wales) recommended more research on the experiences of those subjected to disparities in CAT to address an evidence gap.

### Lived experience research innovation

Lived experience research has exposed injustices, hidden processes and structural mechanisms in studies of black feminism, critical race and intersectional theories.^9^,^11^ Lived experience research is usually undertaken using qualitative methods, which have advanced our understanding of complex mechanisms by which multiple health conditions most affect those at the intersections of ethnicity and sex.^12 13^ These mechanisms include interactions between different vulnerabilities and forms of disadvantage, for example, living in poor places with more crime may be associated with more stretched services as well as greater care needs due to violence and trauma. There are very few qualitative studies of CAT, and none that are experience driven and that capture the experiences of ethnically diverse groups.^14^

However, there are philosophical, theoretical and practical tensions. Lived experience research differs from conventional qualitative research, in that the emphasis is on the personal experiences of the participant and peer researchers interpreting the experience data, rather than the researchers’ primary, secondary and tertiary conceptualisation dominating the findings. Conventional research methods are often not well tailored to specific groups and are often not sufficiently flexible to accommodate the needs of potential participants and support them in and out of research and give them a good experience of research. Thus, conventional research methods tend to under-recruit marginalised groups. Under-representation may be explained by language barriers, fluctuating levels of insight and capacity, fear of losing freedoms and fearful avoidance of services and research institutions. Even if recruited into a research study, people may struggle to verbalise painful memories, especially if the research processes do not adequately support participation of people living with multiple everyday struggles, disabilities, frailties and precarious social situations.

We address these problems by using participatory methods that are particularly valuable to engage marginalised groups. Specifically, photovoice (PV) involves asking participants to take pictures and narrate their meaning. PV is more accessible as a research process for people with varying levels of verbal literacy and disability as their experiences need not be expressed verbally and can be reflected on, and evolved over time.^15^,^18^ The approach disrupts the traditional one-sided relationships between researcher and participant by foregrounding the lived experiences of participants throughout the research process and placing more power in the hands of the participant whose creativity and perspectives are the starting point and remain central; these perspectives can reveal additional topics for further enquiry and recommend improvements in the conduct of research.

PV has not previously been used to inform experience-based intervention design for ethnically diverse patients subjected to CAT.^18 19^ In this study, we use PV and emphasise the *primary experience of participants*, and interpretation by peers and researchers staying close to these experiences. Furthermore, we endeavoured to note, retain and report on *common and uncommon experiences in the data set, as both* are important. Many qualitative research designs tend to report common experiences only, and statistical studies usually report average group effects. However, there may be uncommon experiences from those recruited into research, which have an important bearing or shed light on a hidden problem; and in the population, there may be more people facing those experiences. We also endeavoured to consider *the intersectional position of participants* noting their age group, sex and ethnic group, so that similarities and contrasts by intersectional location were visible.

### Objectives

- Assemble the life and care experiences of racially and ethnically diverse people who experienced CAT.
- Identify biographical, interpersonal, social and service-related influences leading to CAT
- Inform progressive preventive care, policy and legislation.

## Methods

Between July 2021 and April 2022, we recruited participants within 2 years of at least one CAT episode under the powers of the MHA (England and Wales, 1983, 2007; MHA) in one of eight locations in England (Oxford, London, Birmingham, Leeds, Derby, Bradford, Manchester and Lancashire). These venues were chosen to reflect urban and rural areas and ethnic variation of residents. The intention was to ensure representation from different geographical areas with different local service structures and socioeconomic and social contexts. Purposive sampling^20^ ensured participants were from diverse ethnic groups, and representative of a range of ages, sex and specific sections of the MHA (Sections 5 (4), 5 (2), 4, 2, 3, 136); we excluded participants who had only experienced forensic sections. The design ensured an ethnically diverse sample, rather pursue comparison by ethnic groups. We supported participation, for example, by paying for carers. Community organisations and National Health Service (NHS) Trusts assisted. We involved potential participants through coffee mornings, local charities, the National Service Users Network, Non-Governmental Organisations (NGOs), radio channels, and the project’s Public Participant Involvement Research Group (PPIRG) and its networks. The PPIRG included six people (two men, one White British person) who cared for someone who had experienced CAT or had direct experience of CAT. The PPIRG influenced the design, execution and interpretation of the research and was led by a Black British woman with experience of being subjected to CAT.

### PV procedures

The rationale, methods and protocol were published.^21^ Participants took photographs and reflected on and captioned these over three workshops (WS) as set out in box 1.

Box 1 **Photovoice workshops**

**WS1. Induction**

- How to take photographs (inclusive of ethical considerations), what might be photographed, sharing information about the research, seeking consent to participate.

**WS2. Images, captions and experience narratives**

- Participants were given disposable cameras or could use their own cameras, or they could bring other images, or forms of art, song or poetry. Images were shared in person, or by e-mail or the study WhatsApp channel. Individuals chose specific photographs and developed captions and narrative description, guided by *SHOWeD* questions.^48^ SHOWed refers to: show me what See here? what is really Happening here? how does this related to Our lives? Why does this condition Exist; what can you Do about it? We collected demographic information, life events, biographical and compulsory admission and treatment-related information on Roadmaps. These consisted of a black and white drawing of a path, representing the timeline of their life, onto which participants added life events and experiences.

**WS3. Reflection**

- Participants shared the images and narratives with each other and considered: what made a difference to their experiences and what could be done differently? These discussions were captured by audio recorder and transcribed verbatim.

WSs were conducted flexibly, at least a week apart, and in person or online, led by RM with two research support staff (CD and MY) and representatives from the PPIRG and the research team. In-person WS were held in accessible public spaces, such as community centres and art galleries; away from NHS settings wherever possible. We used Padlet**,** a shared online digital canvas, to share ideas and images and record captions for online participants (see padelet.com). Participants received a £15 Amazon voucher to thank them at the end of each WS.

### Main outcomes and measurement: data analysis

In the absence of standardised methods for analysis and reporting image, caption and narrative data,^22^ we used the principles of thematic and polytextual thematic analysis (PTA).^23 24^ PTA refers to taking account of multiple ‘texts’ including creative visual and arts materials alongside written texts.^23 24^ We used *Framework* to organise the images, captions and narrative excerpts in charts (in excel), making the process explicit and transparent, permitting checks for consistency. Framework is a structured process for qualitative data analysis, especially for policy research, including making the analytic process transparent and open to scrutiny and verification; data are often charted to represent the themes, and this permits the data to be compared across different participants and by other relevant characteristics.^25^ The charts are also a convenient way to present summary information to participants, the PPIRG and the research team.

The analysis drew on lived experience approaches to thematic analysis, grounded in critical realistic ontologies that *experience data* reflect meaningful realities in the external world.^26^ In accord with guidance, the analysis deepened knowledge rather than relying only on common themes and inter-rater reliabilities.^26 27^ KB and RM undertook a thematic analysis, using constant comparison.^27^ They independently coded and then cross-checked about 40% of the data following a process of familiarisation, to develop a preliminary inductive coding framework (codes meaning centred interpretive stories). KB completed analysis of the rest of the data until saturation. Reflexivity was considered in all processes, with lived experience interpretation occurring alongside the thinking of researchers. The racially and ethnically diverse research team, including the PPIRG, interpreted the data and critiqued publications. Nonetheless, we were sensitive to the dynamics of race and racism and our respective identities throughout the study and in the analysis and interpretation. KB, a male, Kenyan-born psychiatrist of Punjabi Sikh Indian heritage, holds a research doctorate (MD); RM is a woman of Indian and Irish heritage; she holds a PhD in Psychology. Both have undertaken extensive qualitative research (over 25 and 10 years, respectively). The conclusions were interrogated by investigators and lived experience experts in the research.

## Results

Of 60 recruited participants, 48 completed all three WS. Eight of the non-completers were from the first workshop and did not return images or attend later workshops; four became unwell. There were three broad types of data: (a) demographic and roadmap information, (b) 535 images and captions, (c) 32 hours of recorded narratives (averaging 90 min for online participants and 2.5–3 hours for in-person workshops).

### Demographics

Participants were aged 18–65 (age<20, n=2; 20 to 29 n=12; 30 to 39 n=14; 40 to 49 n=8; 50 to 59 n=12; 60 to 65 n=2; mean=41.1; SD=12.2). There were 24 men and 24 women. Most participants were ethnic minorities (see table 1). From the roadmaps, which were completed without prompting or structured questions, we identified significant levels of multimorbidity (35.2%, n=17), adverse childhood experiences (45.8%, n=22), concerns about impacts of their detention on their children (31.3%, n=15) and police contact (26.8%, n=10). All of these suggest that people who are subjected to CAT have other areas of need including for physical healthcare, their children’s health and that adverse childhood experiences make CAT and police involvement especially traumatic.

**Table 1:** Co-Pact participant demographics (N=48, except were different N specified)

Sex	Male	24	50
	Female	24	50
Ethnicity	WB	11	22.9
	BBC	13	27.1
	BBA	4	8.3
	BB	1	2.1
	IND	1	4.2
	PA	2	18.8
	BRiAS-BANG	9	2.1
	BRiAS	1	8.3
	MIXED Chinese	4	2.1
	MIXED South American	1	2.1
	MIXED	1	2.1
City	Leeds	4	8.3
	Manchester	3	6.3
	Bradford	4	8.3
	Derby	4	8.3
	Birmingham	8	16.7
	London	13	27.1
	Oxford	8	16.7
	Lancashire	4	8.3
MHA section	2	20	43.5
(N=46)	3	21	45.7
	5 (2)	3	6.5
	136	2	4.4
Diagnoses			
Schizophrenia		5	10.4
Schizoaffective		8	16.7
Delusional		1	2.1
Bipolar		14	29.2
Major depression with psychosis		3	6.3
Borderline personality		4	8.3
Cannabis use and psychosis		1	2.1
Major depression without psychosis		2	4.2
Functional psychosis		7	14.6
Acute transient psychosis		2	4.2
Recurrent major depression		1	2.1
Roadmap data			
ACE	Yes	22	45.8
Physical health poor	Yes	17	35.2
Children	Yes	15	31.3
Police path	Yes	10	26.8

BA, Black African; BANG, Bangladeshi; BB, Black British; BBA, Black British African; BBC, Black British Caribbean; BRiAS, British Asian; IND, Indian; MHA, Mental Health Act; PA, Pakistani; WB, White British .

### Overarching synthesis

**Figure 1:**
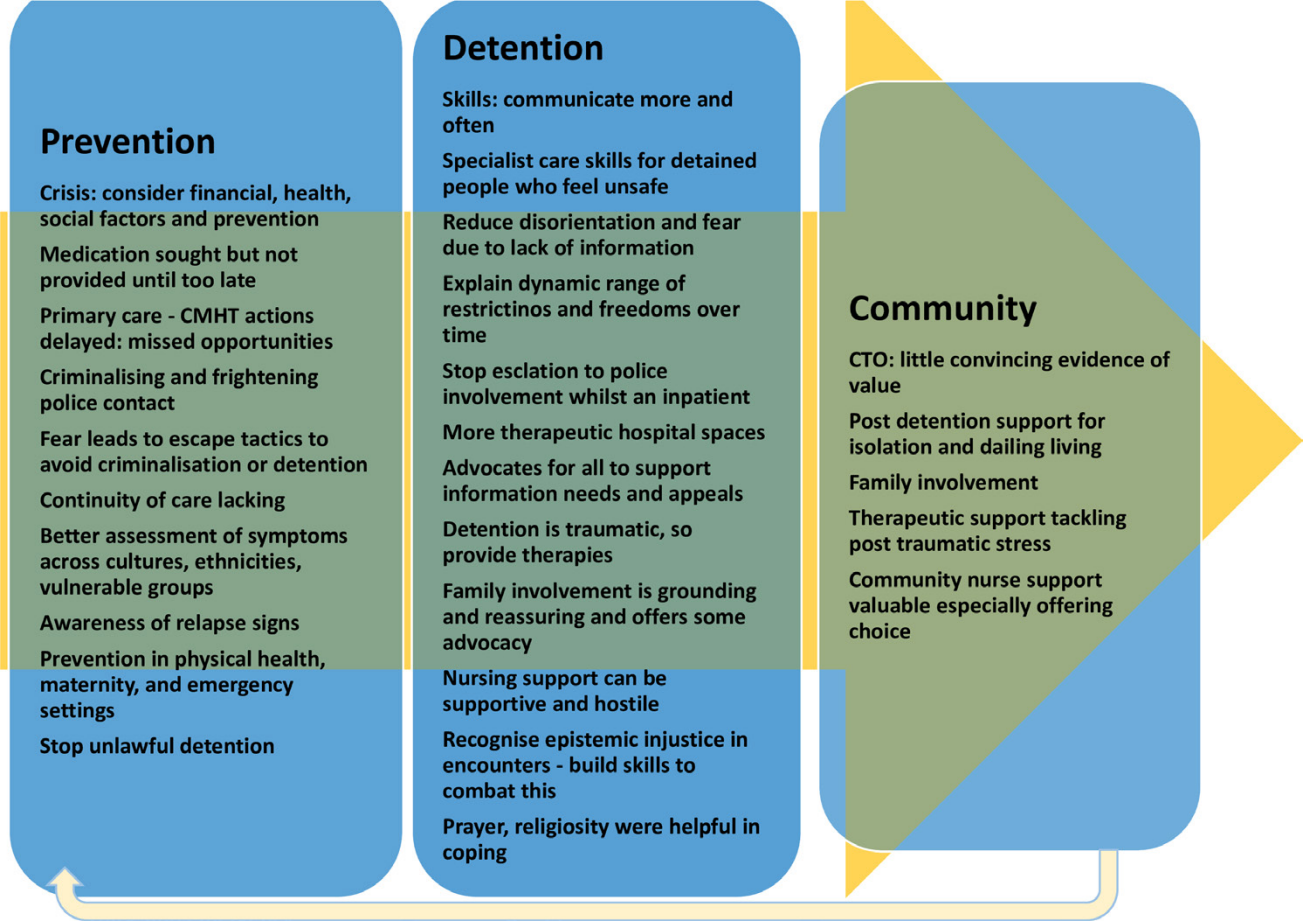
Overall thematic analysis of experience data. ACE, adverse childhood experiences; CMHT, community mental health team; CTO, community treatment order.

First, we report an overall inductive thematic analysis of the transcripts (see figure 1; Online supplemental table 1), with some illustrative images and captions. The full image library is accessible here: https://www.flickr.com/photos/198122100@N06/albums/72177720307479644/.

### Awareness and prevention

Participants were often aware of deterioration in mental health and sought intervention early. Despite this, many received no preventive intervention and were admitted in crisis (see figure 1). Financial difficulties were sometimes the trigger for a relapse and admission, and participants proposed financial interventions could potentially avert stress and CAT.

Online supplemental Image 1: *Banging your head against a brick wall… Sometimes I find mental health care/accessing mental health care can be like hitting your head against a brick wall. Being detained is all well and good… as long as there’s a bed for you. Which often there isn’t. I’ve been on the ‘bed list’ for 5 weeks now (for informal admission this time). Multiple weekly incidents, police and ambulance involvement… and still…. ‘No beds’*. (F, WB, 30-239)

### CAT processes

Some detention decisions were influenced by the availability of beds and not the need for admission. In some sites, people travelled for 4–5 hours and were admitted far from their home. Two participants described being detained and transported in an unmarked van, which felt like ‘being kidnapped’ as there was nothing to indicate these were health service vehicles.

Online supplemental Image 2: *This symbolises the journey of being taken to hospital. The first time I was sectioned I was taken to hospital in a black van by three people not wearing uniforms. I thought I had been kidnapped. It was scary, confusing and lonely. It’s like a journey into the unknown*. (F, 50-59, White British)

**Figure 2:**
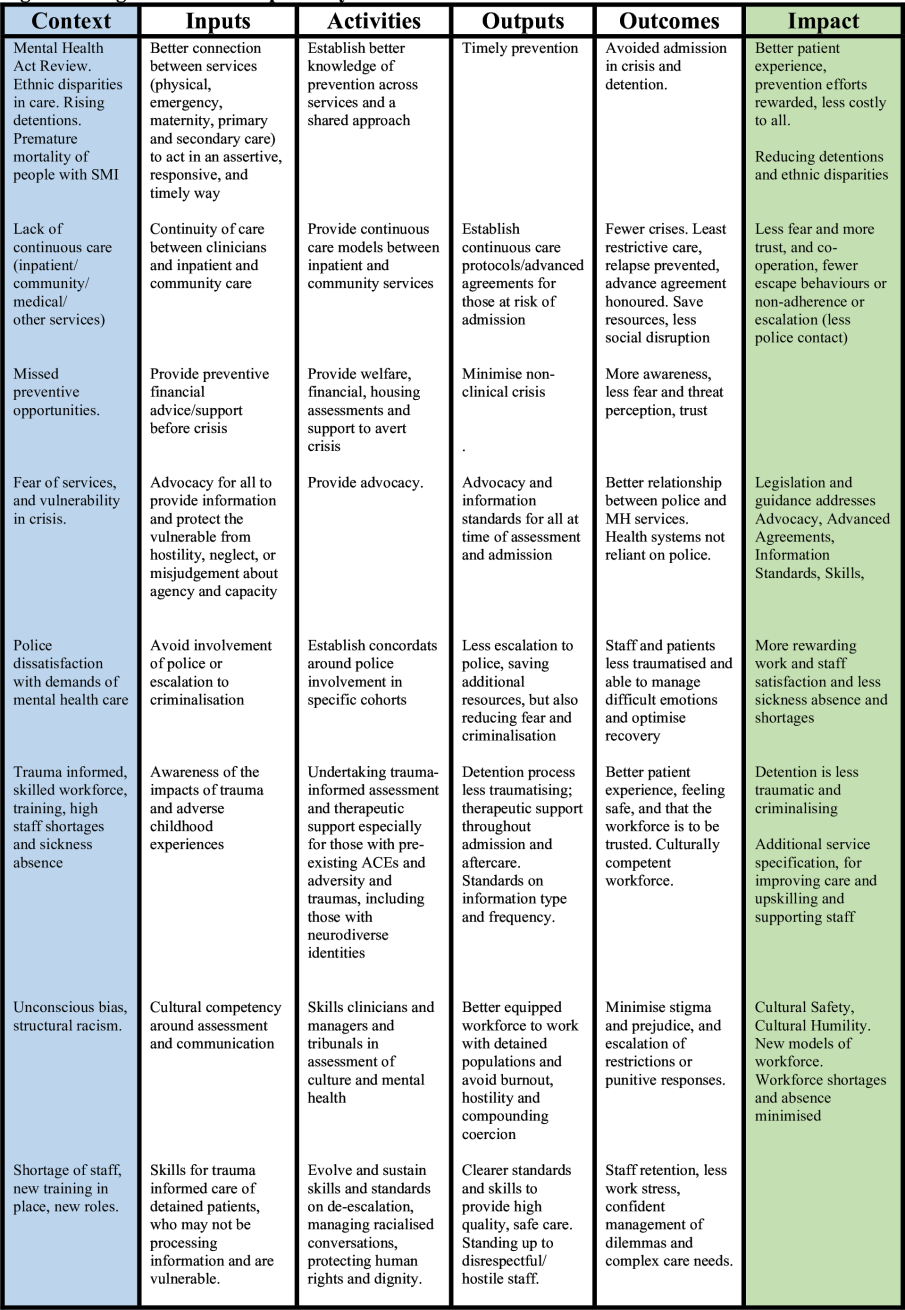
Logic model of potential interventions. ACE, adverse childhood experiences; MH, mental health; SMI, severe mental illness.

### Coercive care and staff interactions

There were strongly held views that practitioners did not have sufficient experience, knowledge or skills to support people in distress at the time of CAT. Participants recognised the challenge of caring for very unwell people and the demands on staff. However, they experienced too much variation in skills (eg, by day and night shifts) and much stigmatising language. Staff responses were sometimes hostile, dehumanising, disrespectful, threatening, openly rude and even aggressive. Participants felt coerced and did not understand why professional standards for care and concern were not evident. In one instance, a person described being forced to have medication as ‘like being raped’.

Online supplemental Image 3: *We do not get care. We are meted out punitive treatment that makes our communities apprehensive about contacting so-called support services in the first place. We are treated more like criminals* (F, 50-50, Mixed African English).

The lack of regular interaction with professionals was a source of dissatisfaction. Communication was often through small openings in doors. The only interactions were to receive medication or record observations. At times of staff shortages, leisure activities were restricted, as risk management was prioritised. The accuracy of medical records was questioned. For example, when secluded participants felt blamed, and the records did not reflect their experiences, despite long periods of detention. One participant described a diagnosis was shared with the employer without permission, resulting in long-standing unemployment.

There was evidence of good practice too. Activities such as painting or quizzes were welcomed and helpful, especially at weekends, which were very quiet. There were positive comments about community nurses acting with compassion and being more flexible than inpatient staff. Specifically, community staff were willing to trial different approaches, whereas inpatient care seemed less flexible and inattentive to needs. Reassuringly, some participants said they trusted staff advice and it was good to know they ‘had my back’.

### Therapeutic environments

Participants felt waking on a ward without explanation was disorientating, especially as professionals wore plain clothes. Consequently, they did not know if they were in a safe place. Participants wanted more information repeated on several occasions, to understand options and what was happening to them. They felt professionals should repeat messages and use different modalities of communication, for example, in induction packs. Participants were uncertain what ‘detention’ meant in terms of which options and choices they had at different points of their hospital stay. Participants received little information about their seclusion. There were few opportunities for exercise or ordinary things. In some environments, the facilities fell short: no bedding, no toilet seat, no daylight, with nursing staff isolated in their station or office. ‘It felt like a zoo’, and so noisy that they could not sleep. It was not perceived to be a good place to recover, although short stays were tolerable.

Online supplemental Image 4: *this picture is about exercise. It’s good to exercise every day. Good for your mental health. But when I was detained I could not exercise for a while*.

*It a good thing to exercise but under detention is impossible* (M, 40–49, Black British).

Families and carers

Families were often not permitted to visit or did not visit. Active involvement of family, friends or carers helped participants feel calmer. Maintaining these relationships restored dignity by reconnecting with their lives outside hospital.

Online supplemental Image 5: *this picture depicts a one to one with my eldest son. It makes me feel like I'm meaningful. We would talk about what goes on for each of us over the past weeks. And what our plans are for the coming weeks* (F, 30–39, Black British Caribbean).

### Specific insights

#### Police

Thirteen participants commented on police involvement (4 women; ethnicity: 4 WB, 2 P, 5 BB, 1 BC, one mixed ethnicity; age: one aged 10–29, two aged 20–29, four aged 30–39, two aged 40–49, three aged 50–59, one aged 60–89). Participants feared escalation and the use of force when in contact with the police. Unexpected police arrivals were distressing and embarrassing: ‘everyone watched’, ‘they drag you out sometimes with handcuffs and kicking’. Participants felt powerless and criminalised and had to keep themselves safe in vulnerable moments. One participant (M, P, 20–29) said he expressed symptoms of mental illness to get to hospital and avoid being criminalised when in contact with the police. Some believed hospital admission for care *required* a police arrest first.

Participants sometimes called the police. For example, when a person feared a house break-in. He feared being kidnapped when asked to go into an ambulance. On refusing, he was arrested, put in a van, taken to the ward, and placed in an isolation without explanations (M, WB, 10-19)^.^ At other times, staff called the police. An inpatient nurse called the police in response to requests for help and medication that persisted for over a half an hour. The participant was then placed in a seclusion room, which was disorientating as there were no windows. She could not tell if it was day or night, and it was cold. Then, she was threatened with being ‘*IM’d’* (intramuscular medication against her will).

There were also positive experiences of police. Participants said the police were generally good at the job and could be ‘more understanding than paramedics’ (F, WB, 30-39). A woman (F, BB, 30-39) was surrounded by police on the way home, after she stopped in a park to ‘rest and get some air’. She had stepped onto train tracks, so in this instance, police intervention was helpful.

#### Preventive actions

Nine people referred to prevention (4 women; ethnicity: 4 WB, 1 P, 2 BB, 1 CB, 1 mixed ethnicity; age: one 10–19, two 20–29, three 30–39, three 50–59). Participants accepted that medication could prevent detention. Some professionals encouraged reflection on signs of relapse to prevent CAT. There were early actions by patients to prevent admission; however, preventive actions took too long to avert a crisis. A woman with a postpartum psychosis had 5–10 min with a midwife during the pandemic. As a single mum with a family history of mental illness, she felt her mental health should have been noticed and the relapse prevented. She was separated from her child for a week due to admission.

#### The dynamics of communication and assessment

Contact with a care coordinator was helpful when there was a trusting relationship (F, P, 20–29). More could have been done to prevent admission when psychosis was emerging (M, WB, 30–39), by the community or primary care team. Another participant (F, BC, 20–29) asserted that the diagnostic and formulation process was not culturally sensitive or precise, with excessive negative reading of experiences and behaviours. Misdiagnosis and misinterpretation were argued to explain higher admission rates for ethnic minorities.

#### Emergency departments

Seeking help from emergency departments was not a good experience (F, WB, 30–39): ‘you’re pushed aside and looked down on’. Contrary to guidance and legislation, some were held in emergency departments despite not being on a section (M, WB, 30–39). Again, some had positive experiences: ‘….security guards, ambulance staff, emergency staff, everybody was lovely’.

#### Feeling safe and advocacy

Isolation and loneliness were common experiences, some wanting more communication and a person with them all the time on the ward, especially in moments of crisis. Isolation in uncomfortable surroundings reinforced a lack of safety. Participants felt unentitled to humane environments and questioned why such conditions were considered reasonable (police cells, seclusion rooms and assessment and inpatient facilities). Advocacy has been proposed as a solution. There were mixed experiences of advocates (3 women; ethnicity: 1 WB, 2 BB, 1 P; age: two 20–29, one 30–39, one 50–59). Some advocates supported the doctor’s position, insisting the patient takes the medication. Some participants felt everyone should have an advocate, because of their ‘state of mind’ when first detained. Advocates helpfully explained participants’ rights and how to get a solicitor, who helped end a section by articulating the participant’s case, organising evidence. Otherwise, the paperwork for an appeal was too burdensome (F, WB, 50–59), especially for a person in distress. Furthermore, advocates reassured participants that someone cared and helped to do what they could not manage alone.

#### Detention trauma

Eight participants (5 women; ethnicity: 4 WP, 1 Ba, 1 In, 1 BB, 1 P; age: two 20–29, two 30–39, two 40–49, one 50–59, one 60–69) said the process of CAT was traumatic. They talked of isolation, loneliness, losing control over everyday decisions and feeling distressed. Several spoke of ‘being broken’ by the experience rather than seeing it as helpful or restorative. For those with pre-existing post-traumatic symptoms, additional distressing events were felt as even more traumatic.

#### Community support

Leaving hospital was liberating (F, mixed race, 50–59; F, P, 20–29), giving access to gyms and shops. However, ‘adjusting back into living on your own in a quiet place^’^ was difficult in contrast to the ‘noise and number of interactions on a ward’. There was a need for structure outside the hospital, by joining health groups in the community; the only formal advice on leaving was around taking medication ^(F, WB, 30-39)^. Participants felt being discharged without carers or a network of community support meant people became unwell again and returned to hospital.

#### Religion, race and ethnicity

Religious practice was helpful, for example, ‘praying when ill’ (F, P, 20–29; M, BB, 40–49). Yet there was often no place to do so on the ward. A multifaith room in one site was highly praised as an example of good practice. There were positive experiences of chaplains alongside apprehension about expectations of orthodoxy (F, WB, 50–59). One chaplain helpfully spent an hour with a participant, but variations between hospitals and over time were a concern. The participant was a Buddhist, and there was no religion-specific support for her. Another (F, British Asian, 30–39), a Muslim, was not given choice of appropriate foods.

A patient with a strong Caribbean accent was ignored and misunderstood as aggressive. One participant (M, BC, 50–59) felt black people were over-admitted rather being given home treatment. On multiethnic inpatient units, people felt they had to hide concerns about ethnicity; for example, staff from India speaking only to men, and other staff refusing to speak English. Racialised explanations arose within staff and patient groups and between them.^9^ Victimisation (by staff) of the same person was witnessed, while others were left alone even when they infringed rules. Positively, some noted (M, WB, 30–39) that there were staff of many ethnicities, and they were caring; whereas other participants found this to be a source of tension and potential conflict. Yet, having staff who looked like the patient was helpful (F, BA, 20–29).

## Discussion

Our findings expand previous recommendations^28^ and identify new social and psychological and practice-related mechanisms leading to CAT and corresponding preventive opportunities. The participants wanted better information, psychological safety, trauma-informed therapeutic actions from inpatient care and into the community, family involvement, recognition of adverse life experiences and awareness of the impact of social contexts and biographies. More information was sought by patients subjected to CAT; this is especially important as there is evidence that information processing can be impaired in threatening situations as well as in psychosis requiring more intensive and tailored support.^29^

### Epistemic injustice

We emphasised lived experience narratives hold important information. There was evidence of epistemic injustice, one element of which is testimonial injustice,^30^ the discrediting of information based on specific characteristics; in this instance, related to demographics, racialisation, ethnicity, diagnoses of mental illness and loss of agency due to CAT. The other element of epistemic injustice (which we discuss later) is hermeneutic injustice, which refers to a lack of conceptual tools and resources, even a language to articulate concerns; this includes systems of knowledge production. Participants communicated instances of hostility and discrediting of their views. In response to requests for help, participants were ignored leading to relapse and ultimately CAT. Legislation alone will not address epistemic injustice, rather we need alternatives to CAT, long-term investment, better professional skills and care standards, supported by clearer intersectoral policies.^8^

### Specialist communication and care skills

The racialisation of patient-staff interactions can add to feelings of threat and undermine therapeutics; many participants described dehumanising mistreatment, potentially due to racialisation, but ultimately due to a lack of care skills or ability to talk about race. The threat of escalation and the use of force made people feel unsafe and less able to shape their care. Many participants had experienced childhood traumas making CAT and poor communication an even more intense threat experience. Shared decision-making can be useful^28^ but needs better skills standards for the care of people experiencing CAT.

The findings suggest that we need new prevention models and commensurate trauma-informed assessment and care across community, inpatient, general medical, social and specialist care like maternity. Advanced choice agreements, also known as advanced directives, could help with relapse prevention and build trust if inserted into discharge planning.^30^ Integrated care systems and more generalists rather than specialists might help tackle stigma and provide more cohesive social and healthcare systems for prevention.

### Police involvement

Police involvement in mental healthcare is hotly debated.^31 32^ CAT can be felt as deeply racialised and difficult to separate from racism in society.^33 34^ Police involvement and criminal justice pathways in mental healthcare have been questioned for some time.^35 36^ Efforts to avoid coercive escalation and police involvement may improve trust in healthcare agencies.^37^

### Future prevention research

Future research practices (as well as services) will need to reflect more complex eco-social models of illness and consider the intersections of racism, migration, religion and complex trauma, alongside age, sex and place.^19^ Further research and evaluation should encompass primary care, specialist mental healthcare, maternal care, emergency departments and social supports and public venues.^38^

From these findings, we developed a logic model (see figure 2) summarising preventive interventions that could change care experiences and outcomes. This logic model can inform the development of a comprehensive set of interventions to reduce CAT and improve the experiences of people subjected to CAT. Important areas for further work include developing and testing financial and social interventions among those facing hardship and adversity to reduce distress that leads to crisis.^39^ Trauma-informed therapies require further development and testing to prevent future CAT episodes and improve recovery. Although racial trauma is recognised,^40^ the true impact of CAT on recovery, agency and confidence warrants more research, not least in the context of historical racialisation and criminalisation of the mentally ill.^41^ For example, material resources and social ideas about race can also lead to racialisation in systems.^42^ The findings from future research should inform the further iteration of the logic model, providing more detail on potential mechanisms that might be targeted for preventive interventions.

### Strengths and limitations

Epistemic injustice includes hermeneutics, which shines a light on how our structures of knowledge production (research), providing public services, and solving problems may themselves be perpetuating inequalities.^30^ Participatory methods helpfully challenge structures of knowledge production, disrupting traditional power relationships between patients and practitioners.^43^ We brought PV and participatory processes together to assemble CAT experiences and engage people that would not otherwise participate in research. Furthermore, we evolved appropriate analytic methods drawing on PTA and Framework.

Our lived experience approach explicitly identified important issues to combat epistemic injustices emerging from conventional methods.^11^ Racially and ethnically diverse peer researchers and lived experience experts (DJ and SB, NC and MM as members of PPIRG members) were equal partners in all stages of design and execution, communications, analysis, interpretation and dissemination, helping to overcome epistemic injustice in research processes.

We experienced recruitment challenges due to a lack of complete ethnicity and section data in some recruitment sites. Consequently, the number in each locality was not very high, yet representation across areas still serves to ensure different social and area contexts and services were considered. During the post-COVID recovery period, there were also staff shortages and pressures on clinical time. Some services enforced COVID policies that prevented community-based recruitment and data collection. Our data initially recruited 60 people, of whom 48 produced narratives and images; this dataset is still one of the largest that focus explicitly on CAT, and on intersectional lived experiences of racially and ethnically diverse people.^14 44^ The depth of information reflects the power of PV to enable people to participate in research. Sample size in qualitative research is driven by study purpose, depth and volume of collected data. Although samples of 12 may be sufficient for saturation among homogenous populations,^45^ our samples were determined by notions of *information power*^46^ and sample *adequacy*,^47^ assessed during and after the study.

## Conclusions and relevance

These lived experience data show many missed opportunities for prevention. We demonstrated testimonial and hermeneutic forms of epistemic injustice, and we propose the implementation and evaluation of lived experience informed preventive interventions and research recommendations.

## Supplementary material

10.1136/bmjment-2025-301655 online supplemental file 1

## Data Availability

Data are available upon reasonable request.
